# Impact of Chronic Viral Infection on T-Cell Dependent Humoral Immune Response

**DOI:** 10.3389/fimmu.2017.01434

**Published:** 2017-10-31

**Authors:** Stéphane Rodriguez, Mikaël Roussel, Karin Tarte, Patricia Amé-Thomas

**Affiliations:** ^1^UMR U1236, INSERM, Université de Rennes 1, Etablissement Français du Sang Bretagne, Equipe labellisée Ligue Contre le Cancer, LabEx IGO, Rennes, France; ^2^Centre Hospitalier Universitaire de Rennes, pôle Biologie, Rennes, France

**Keywords:** germinal center, T follicular helper cells, B cells, fibroblastic reticular cells, follicular dendritic cells, lymphoma, Epstein–Barr virus, human immunodeficiency virus

## Abstract

During the last decades, considerable efforts have been done to decipher mechanisms supported by microorganisms or viruses involved in the development, differentiation, and function of immune cells. Pathogens and their associated secretome as well as the continuous inflammation observed in chronic infection are shaping both innate and adaptive immunity. Secondary lymphoid organs are functional structures ensuring the mounting of adaptive immune response against microorganisms and viruses. Inside these organs, germinal centers (GCs) are the specialized sites where mature B-cell differentiation occurs leading to the release of high-affinity immunoglobulin (Ig)-secreting cells. Different steps are critical to complete B-cell differentiation process, including proliferation, somatic hypermutations in Ig variable genes, affinity-based selection, and class switch recombination. All these steps require intense interactions with cognate CD4^+^ helper T cells belonging to follicular helper lineage. Interestingly, pathogens can disturb this subtle machinery affecting the classical adaptive immune response. In this review, we describe how viruses could act directly on GC B cells, either through B-cell infection or by their contribution to B-cell cancer development and maintenance. In addition, we depict the indirect impact of viruses on B-cell response through infection of GC T cells and stromal cells, leading to immune response modulation.

## Introduction

Defense against pathogens involve an immediate innate immune response, going along with the establishment of a long-lasting adaptive immune response. Secondary lymphoid organs (SLOs), such as lymph nodes, spleen, tonsils, or mucosal associated lymphoid tissues, are functional structures widely dispersed in the entire body, and ensuring adaptive immune response initiation (1). Indeed, they are meeting points for antigens and lymphocytes allowing the development of memory B and T cells, as well as the differentiation of specialized antibody-secreting cells. The architecture of these lymphoid structures is highly organized, with well-delimited B- and T-cell zones of maturation delineated by specific stromal cell subsets: fibroblastic reticular cells (FRCs) in T-cell areas and follicular dendritic cells (FDCs) in B-cell zones (2). Mature B cells at their different stages of maturation are predominantly localized in primary or secondary follicles. Different steps are critical to allow complete B-cell differentiation into long-lived plasma cells secreting high-affinity antibodies mediating the humoral immune response. These steps include proliferation, immunoglobulin (Ig) gene modifications, cell selection based on affinity for antigen, and require intense interactions with specific CD4^+^ helper T cells belonging to follicular helper lineage. Nevertheless, some viruses can directly or indirectly disturb this subtle machinery affecting the T-cell dependent humoral immune response.

## Germinal Center (GC) Reaction

**Figure 1:**
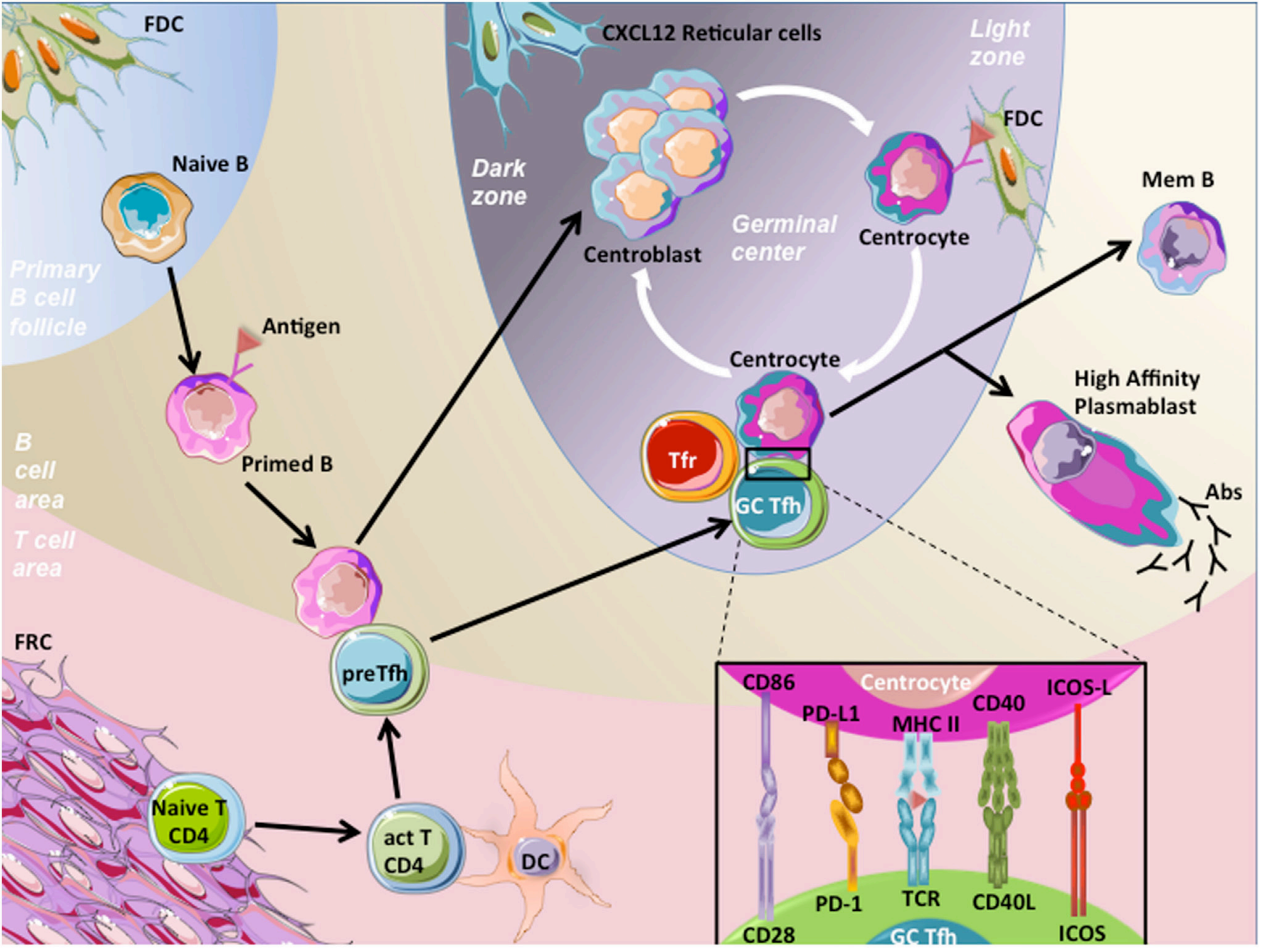
Germinal center (GC) reaction. Naive B cells patrol within the B-cell area in order to encounter specific antigens and become activated through their B-cell receptor (BCR) engagement. Then, primed B cells migrate at the T–B border, and meet pre-T follicular helper (pre-Tfh) cells, which are CD4^+^ T cells that have been previously activated (act T CD4) by processed antigens presented by mature dendritic cells (DC) in T-cell area. This cognate interaction between pre-Tfh cells and primed B cells involved the recognition of processed antigens presented by the primed B cells to pre-Tfh cells. Following this interaction, both cell types downregulate EBI2 and CCR7 and increase BCL-6 expression, a prerequisite to cell migration within follicles, initiation of the GC reaction, and maturation of B cells and pre-Tfh cells in centroblasts and germinal center T follicular helper cells (GC Tfh), respectively. Centroblasts, displaying membrane CXCR4 expression, localize in close contact with CXCL12-expressing reticular cells in the dark zone of GC, proliferate, and undergo somatic hypermutations. This latter process results in generation of centrocytes harboring BCRs with variable affinity for the stimulating antigen, localized in the light zone. A selection step driven by follicular dendritic cells (FDC) occurs, in order to choose centrocytes with a high-affinity BCRs. Centrocytes are non-proliferative cells prone to die unless rescued by their interaction with GC Tfh cells. This interaction involves the presentation by centrocytes of processed Ag in CMH-II to T-cell receptors (TCR) of GC Tfh cells. In addition, centrocytes interact with GC Tfh cells through co-stimulatory molecules, such as CD86/C28, PD-L1/PD-1, CD40/CD40L, and ICOS-ligand (ICOS-L)/ICOS. This interaction results in B-cell activation of pro-survival pathways and drives centrocytes to undergo class switch recombination. Thereafter, B cells leave follicles and differentiate either into circulating memory B (mem B) cells or long-lived plasma cells secreting high-affinity antibodies (IgG, IgA, or IgE). GC Tfh cells also egress and become circulating Tfh cells. One mechanism involved in the control of humoral response is related to the inhibitory action of specialized cells termed T follicular regulatory T cells (Tfr). Tfr cells are functional regulatory T cells localized in follicles and represent one of the mechanisms controlling the magnitude of the GC response.

B-cell maturation begins in SLOs, where trafficking naive B cells recognized native antigens through their B-cell receptors (BCR), the membrane form of Igs. To continue their maturation after BCR engagement, these activated B cells need a cognate interaction with pre-activated CD4^+^ T cells. These CD4^+^ T cells present at the T-B border and providing B cell help are named pre-T follicular helper (pre-Tfh) cells and engage immunological synapse, involving various stimulatory molecules, with B cells. After this activation step, part of these B cells differentiates into extra-follicular short-lived plasma cells secreting low affinity antibodies. Alternatively, T cells and B cells enter the follicle by downregulating EBI2 and CCR7 expression (3, 4), allowing the beginning of the GC reaction with maturation of B cells into GC B cells and T cells into mature T follicular helper (Tfh) cells (5). Highly proliferating GC B cells decrease their membrane BCR expression and undergo somatic hypermutations consisting in random point modifications introduced into the antigen binding regions of BCR genes, and allowing BCR affinity modulation. This results in generation of daughter cells harboring BCR with a variable affinity for the stimulating antigen. A selection step follows with the aim of essentially keeping GC B cells with a high-affinity BCR (6). FDCs harbor at their membrane immune complexes containing antigens, which are grabbed by GC B cells with high BCR affinity. Such GC B cells then present antigen-derived peptides to cognate mature GC Tfh cells, which in turn provide them with survival signals and trigger class switch recombination, resulting in the modification of Ig class. Noteworthy, a part of selected GC B cells can be addressed for further expansion and somatic hypermutations (7, 8). Thereafter, GC B cells leave the follicles and differentiate either into circulating memory B cells, or plasmablasts and subsequently long-lived plasma cells secreting high-affinity IgG, IgA, or IgE (Figure 1).

## GC B Cells

Germinal center B cells are mature B cells with differentiation process in progress, leading to high-affinity memory B cell and antibody-secreting cell production. GCs can be spatially segregated in two distinct compartments: the dark zone and the light zone. In the dark zone, GC B cells termed centroblasts are highly proliferative and undergo somatic hypermutations. By contrast, centrocytes, or GC B cells from the light zone, are non-proliferative cells prone to die unless rescued by the interaction with FDCs and Tfh cells. In addition, centrocytes undergo class switch recombination process (9). Centroblast and centrocyte Ig gene modification processes orchestrated by AID (10) favor genetic instability with increased risk of transforming event acquisition (11). Both of these GC B-cell compartments highly express BCL-6, CXCR5, CD10, and CD21, but can be discriminated otherwise. Centroblasts highly express the chemokine receptor CXCR4 at the cell surface, allowing their localization in the dark zone where CXCL12-producing stromal cells are localized (12, 13). Centrocytes downregulate CXCR4, allowing their migration to the light zone, and display a CD83^hi^ CD40^+^ phenotype facilitating cognate interactions with Tfh cells (14). GC B cells are prone to apoptosis and express several pro-apoptotic molecules such as Fas (CD95).

## Non-B Cells Involved in the GC Response

### T Follicular Helper Cells

T follicular helper cells have been recently described as a *bona fide* lineage of memory CD4^+^ helper T cells driven by the transcription factor BCL-6 and specialized in helping the production of high-affinity and class-switched memory B cells and antibody-secreting cells (15–17). Tfh cells are characterized by high expression of program death-1 (PD-1) and the chemokine receptor CXCR5. In association with a low expression of CCR7, their CXCR5^hi^ phenotype allows Tfh cell localization in B-cell follicles. Tfh cells also highly express ICOS, unlike the TBET, GATA3, RORc/RORγt, and Foxp3 transcription factors specific for Th1, Th2, Th17, and regulatory T (Treg) cells, respectively (17, 18). Nevertheless, they have the capacity to synthetize cytokines related to these other helper T-cell lineages, such as IFN-γ, TNF-α, IL-2, IL-4, and IL-17 (19, 20). They also shared the expression of IL-21 with Th17 cells, and human Tfh cells specifically secrete the CXCR5 ligand CXCL13 (19). IL-21 plays a predominant role in the regulation of GC responses and B-cell differentiation (21, 22). Cytokine secretion profile is not uniform at the single cell level. In agreement, the whole Tfh cell population is more heterogeneous than previously assumed and gathers several Tfh cell subsets providing differential help to GC B cells (23). In SLOs, pre-Tfh cells are localized at the T-B border, whereas mature Tfh cells are localized inside the GC, establishing immunological synpases with centrocytes (Figure 1).

More recently, a circulating counterpart of these cells has been described in blood (24). Circulating or peripheral Tfh cells are memory CD4^+^ helper T cells defined by the expression of CXCR5, but at lower level than in SLOs. They are generally also defined as PD-1^+^ CCR7^low^ and can express ICOS (25), but a strict phenotype is not currently consensual. Circulating Tfh cells are functionally defined by the help they can provide to B-cell differentiation into antibody-secreting cells *in vitro*. However, circulating Tfh cells and Tfh cells from SLOs also distinguish clearly by the expression level of BCL-6 that is poorly detectable by flow cytometry in circulating Tfh cells (26).

### T Follicular Regulatory (Tfr) Cells

T follicular regulatory cells have been first described more than 10 years ago as Foxp3^+^ cells within human tonsil GCs (27), but their extended features were further demonstrated in mice (28–30). Tfr cells are functional regulatory T cells localized in GCs and represent one of the mechanisms controlling the magnitude of the GC response after immunization. Tfr cells were more likely to control the function and output of an established GC, with extra-follicular Treg cells controlling the initiation of the GC (31). In addition, Tfr cells have been demonstrated to be implicated in the control of humoral autoimmunity in mice (32, 33). Like other Treg cells, they express inhibitory molecules such as GITR or CTLA-4. However, they share CXCR5, PD-1, and ICOS expression with Tfh cells, but do not secrete B-cell helper cytokines IL-21 and IL-4. Finally, they co-express BCL-6 and FOXP3 transcription factors specific of Tfh cell and Treg cell lineages, respectively (18, 28). Recently, it has been demonstrated that they can originate either from thymic Treg cells or from CD4^+^ activated T cells specific from the immunizing antigen (34).

### Stromal Cells

Different stromal cells derived from non-hematopoietic precursors of mesenchymal origin co-exist in SLOs and reside in different areas. Among them, two subsets are of interest in this review: FRCs occupying T-cell areas and FDCs within B-cell zones (35).

Fibroblastic reticular cells are specialized myofibroblasts organized as an intricate tridimensional network allowing structural organization of SLOs (13). FRCs are involved in immune cell recruitment in SLOs through the secretion of CCL19 and CCL21, the two ligands of CCR7. Naive T cells and dendritic cells (DCs) are in constant contact with FRCs within T-cell area and migrate along the FRC network. Furthermore, FRCs promote T-cell (36), DC (37) and B-cell (38, 39) survival.

Follicular dendritic cells are the lymphoid stromal cell subset involved in recruitment of B cells and Tfh cells in follicles through CXCL13 secretion. They are absolutely required for GC maintenance and B-cell retention (40). One of their key functions is the capacity to recycle for long time native antigens captured as complement-coated immune complexes, allowing their presentation for the GC B-cell selection step (41).

## Modulation of Humoral Immune Response by Viral Infection of GC B Cells

**Figure 2:**
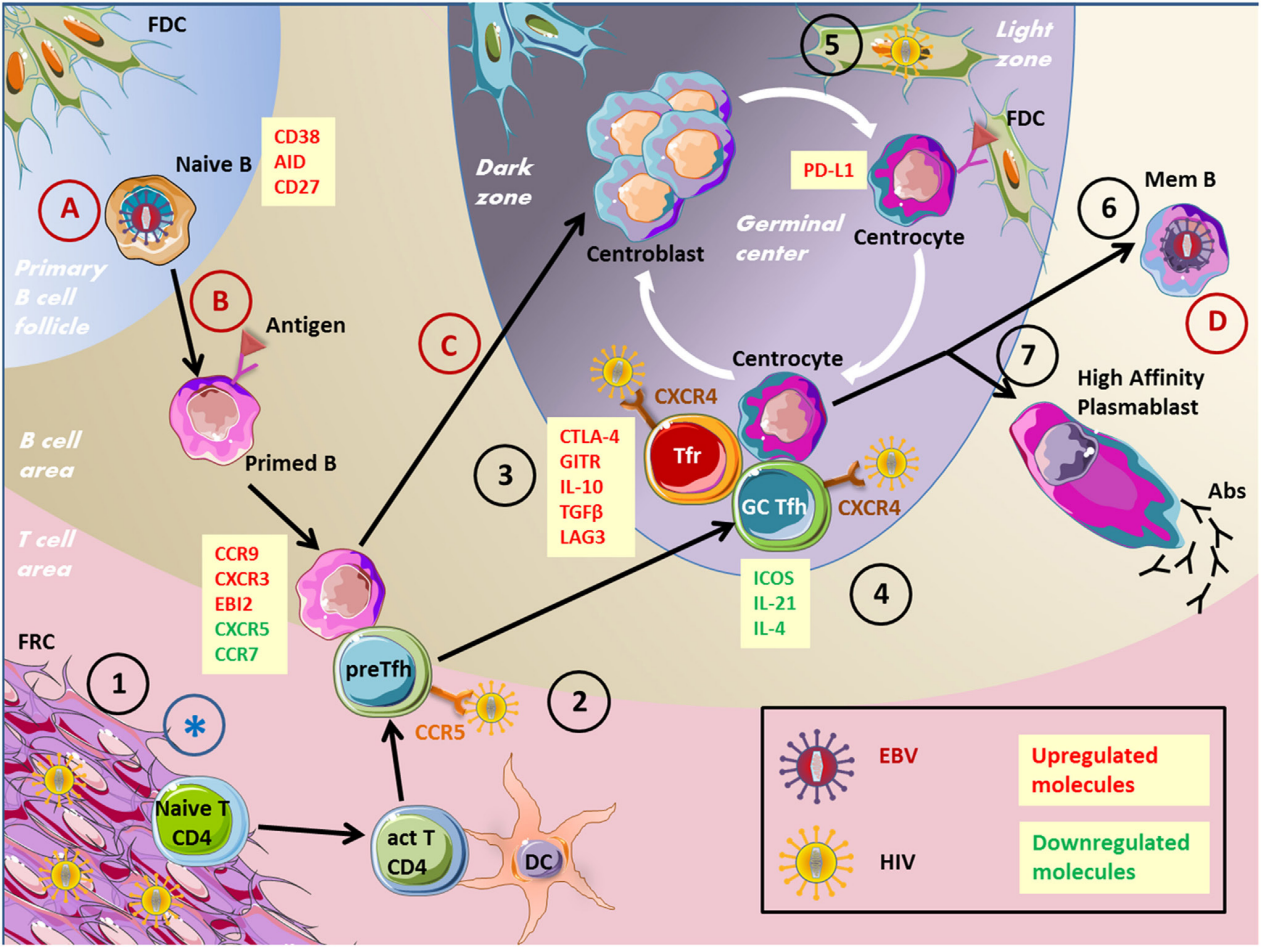
Impact of viruses on the GC reaction. This figure depicts several virus actions on cells involved in the humoral immune response. EBV, Epstein–Barr virus; HIV, human immunodeficiency virus; GC, germinal center; FRC, fibroblastic reticular cells; Tfr, T follicular regulatory cells; Tfh, T follicular helper cells; BnAbs, broadly neutralizing antibodies; Abs, antibodies. 1. HIV stimulates collagen deposition by FRCs, impairing naïve CD4^+^ T cells to access survival signals. 2. Pre-Tfh cells have been found permissive to HIV infection. 3. Tfr cell frequency is increased during HIV infection. 4. HIV persist in Tfh cells, Tfh cells accumulate but are not effective. 5. FDCs are a source of HIV infection for T cells without being infected. 6. Memory B-cell compartment is decreased in HIV patients because of defective Tfh cell help. 7. Impaired specific humoral immune response and hypergammaglobulinemia in HIV patients. (A) EBV infects naïve B cells. (B) EBV drives B-cell proliferation and the expression of differentiation markers. (C) EBV impairs GC entry. (D) EBV persists in memory B cells. *FRC network destruction by several viruses inducing an SLO disorganization.

The principal agent infecting mature B cells is Epstein–Barr virus (EBV) belonging to *Herpesviridae* family (Figure 2). EBV infects the vast majority of humans principally during childhood and persists mostly in latent form throughout life. EBV primary infection is transmitted through saliva exchange before virus entry into epithelial cells. There, virus begins a replication phase through lytic cycles and, therefore, infects B cells through exosome production (42). Once in B cells, linear viral genome circularizes and remains latent as episome within the nucleus. Three types of EBV latency are described, each characterized by the expression of part (type I latency) or all (type III latency) viral proteins, including six EBV nuclear antigens (EBNAs) and three latent membrane proteins (LMP1, LMP2A, and LMP2B). Lytic phase and type III EBV latency are linked to higher immunogenicity and viral replication, while the lack of viral protein expression observed in type I latency is presumed as responsible of the impaired immune recognition allowing virus maintenance. Importantly, GC B cells express a more restricted number of viral proteins, limited to EBNA1, LMP1, and LMP2 (latency II).

Several proteins encoded by EBV genome cross-react with signaling pathways involved in B-cell proliferation and survival. LMP1 is a trans-membrane protein considered as a CD40 mimicry as they share several downstream signaling molecules such as tumor necrosis factor receptor-associated factors, which activate NFκB (43), JAK/STAT, PI3K (44), and MAPK (45) pathways. LMP2A, which is expressed together with LMP1, mimics BCR functions allowing B-cell survival of non-expressing BCR cells isolated from adenoid GC B cells (46). Moreover, this ITAM-containing protein allows the recruitment of the downstream signaling molecules Syk, Cbl, PLCγ2, and p85 (47, 48). These studies performed *in vitro* or with transgenic mice ascribed robust signaling to LMP1 and LMP2, considering that they are able to drive activation and survival in the absence of antigen by hijacking BCR and CD40 signals, and even rescue BCR-defective B cells (43, 49–52). Roughan and Thorley-Lawson showed that *in vivo* EBV-infected cells express LMP1, LMP2, and EBNA1Q-K (the default latency program) but the role of LMP1 and LMP2 is probably less crucial than assumed *in vitro*, perhaps only supplementing physiologic signals to provide a survival advantage in the highly competitive environment of the GC (53). Of note, GC B cells latently infected with EBV undergo *in vivo* intensive proliferation without expansion, due to death or egress process (54).

Epstein–Barr virus persists in circulating memory B cells (55). Controversial studies discussed about direct *in vivo* EBV infection of GC B cells, or GC differentiation access of infected naive B cells. It has been suggested that EBV initially affect naive B cells before experiencing the GC response and preferentially become memory B cells rather than antibody-secreting cells (55). Nevertheless, transgenic mice model of LMP1-expressing B cells revealed that LMP1 induces proliferation and extra-follicular differentiation into antibody-secreting cells and blocks GC engagement (43). *In vitro*, EBV is able to efficiently infect resting naive B cells, leading to continuously proliferating lymphoblastoid cell line establishment. In addition, EBV can also infect GC B cells and rescue them from apoptosis (56). *In vivo*, immunohistochemistry studies revealed that the majority of EBV-infected cells are found within the interfollicular zone of lymph nodes, and only few EBV-infected cells are localized within GCs of healthy carriers of the virus, and infectious mononucleosis patients (53, 57).

Expression of typical GC B-cell markers CD38, CD77, and AID, as well as the activated/memory B-cell marker CD27 in naive B cells transformed *in vitro* by EBV suggest a GC B-cell commitment. Nevertheless, these expressions were lower than in *ex vivo* sorted GC B cells. In addition, EBV transformed GC B cells show a downregulation of CD77, BCL-6, and CD79b, compared to non-transformed GC B cells (58). It has been shown that LMP1 downregulated BCL-6 expression in B-cell lines *in vitro* (49), whereas BCL-6 was proposed to be upregulated *in vivo* in GC B cells in the presence of LMP1 (53). These discrepancies highlight the limitations of lymphoblastoid cell lines as a model of *in vivo* EBV biology.

Mutational status of EBV-infected circulating memory B cells showed that they exhibited similar levels of somatic hypermutations than non-infected memory B cells, evidencing a differentiation step within GCs (59, 60). Most EBV^+^ GC B cells belonged to clones of cells harboring somatically mutated V gene rearrangements. Nevertheless, EBV^+^ GC B cells proliferate without ongoing somatic hypermutations, in contrast with uninfected B cells in the same microenvironment during infectious mononucleosis (57).

B-cell recruitment and migratory capacity into SLOs may also be influenced by EBV infection. Gene expression analysis of *in vitro* EBV-infected tonsillar B cells demonstrated a CXCR5 downregulation while CCR9 and CXCR3 were upregulated (61). Noteworthy, CCR7, which is important for migration into SLOs, was also downregulated in these B cells. Immunohistochemistry reports showed that CD10^+^ cells infected with EBV in tonsils of healthy carriers classically expressed CXCR4 and CXCR5 and displayed low CCR7 expression (53). Furthermore, EBI2, which was originally found as strongly upregulated in EBV-infected Burkitt’s lymphoma B cells together with CCR7, plays a critical role in the regulation of B cells positioning within the GCs. EBI2 is a GPCR signaling through G_αi_, like most chemokine receptors. Its expression is physiologically high in naive B cells, transiently upregulated following BCR stimulation and repressed by BCL-6 during GC B-cell differentiation. Indeed, low CCR7 and EBI2 expression levels are required to guide B cells into follicles where they differentiate into GC B cells (3). B cells *in vitro* infected with EBV show an EBI2 upregulation both in lytic and latent phases (62, 63). Consequently, EBI2 high expression in EBV-infected B cells might favor a decreased GC formation.

To illustrate the impact of EBV proteins during autoimmune diseases, we could focus on two mouse models based on LMP2A and LMP1 expression. Minamitani et al. demonstrated in mice with ectopic B-cell expression of the EBV related protein LMP2A an increased B-cell differentiation in short-lived plasmablasts correlated with an enhanced production of low-affinity antibodies. Moreover, they demonstrated that these plasmablasts are more prone to produce anti ds-DNA or anti-cardiolipin antibodies associated with a glomerulonephritis typical of autoimmune diseases like systemic lupus erythematosus (SLE) (64). Another transgenic mouse model (mCD40-LMP-1) with B cells expressing a chimeric molecule comprising CD40 extracellular and LMP-1 intracellular domains and further crossed with lupus prone mice (B6.Sle1) demonstrated an exacerbation of the disease (65). These mice spontaneously developed GCs, had enlarged lymphoid organs, higher titer of anti-histone antibodies and signs of kidney pathology resulting in an acceleration of the disease. The same mCD40-LMP-1 mice crossed with rheumatoid arthritis mouse model also demonstrated a pathology worsening (66). In human, although EBV and SLE have been correlated, EBV reactivation is likely following SLE flare. However, more longitudinal studies are required to decipher their tight association (67).

Altogether, these reports demonstrate that the impact of EBV on B-cell positioning, proliferation, and survival argues for a disturbed GC reaction and a modified humoral response.

## Modulation of Humoral Immune Response by Viral Infection of GC Non-B Cells

### T Follicular Cell Infection

Recently, Tfh cells have been described as sources of replication of competent human immunodeficiency virus-1 (HIV-1), serving as a significant cellular reservoir (Figure 2). HIV is a lentivirus leading to acquired immunodeficiency syndrome characterized by opportunistic infection and subsequent patient death. HIV is a single-strand RNA virus, which is converted to double-strand DNA by reverse transcription after target cell entry through chemokine receptors. Then, viral genome is integrated to cell DNA through a virally encoded integrase. It has been demonstrated that HIV-1 replication is concentrated within CD4^+^ T cells localized in lymph node follicles during the asymptomatic phase of the disease. In accordance, Tfh cells were demonstrated as highly permissive to HIV infection and, therefore, constitute an important virus reservoir (68). CXCR4 was considered as the major route for HIV entry in Tfh cells. Nevertheless, CCR5 expressed by pre-Tfh cells constitutes an additional mechanism of Tfh cell infection (69).

In contrast to the depletion of activated memory CD4^+^ T cell associated with disease progression in untreated patients, total and HIV-specific Tfh cell accumulation is observed in chronic infection, associated with a skewing of the B-cell population toward GC B cells. Interestingly, Tfh cell frequency is positively correlated with GC B-cell proportion in chronically HIV-infected patients (70).

T follicular helper cell compartment is a heterogeneous population gathering cells harboring Th1, Th2, or Th17 phenotype depending on their cytokine secretion profile. In simian immunodeficiency virus chronic infection, Tfh cell polarization was demonstrated to be biased toward Th1 phenotype, with a majority of Tfh cells expressing CXCR3 and producing IFN-γ and IL-21 (71). Interestingly, CXCR3^+^ Tfh cells comprised a subset of CCR5^+^ cells and contained more copies of SIV DNA than their CXCR3^-^ counterpart. Finally, CXCR3^+^ Tfh cells could be in part responsible for Tfh cell accumulation and sustained GC reaction due to their excessive IFN-γ secretion (71, 72).

Despite germinal center T follicular helper cells and B-cell accumulation, HIV-1 infected individuals display impaired antigen-specific humoral immunity and poor vaccine response, associated with loss of memory B cell subsets correlated with disease progression (73–75). This impaired humoral immune response could be explained by inadequate B-cell help provided by Tfh cells. Indeed, Tfh and GC B-cell co-cultures resulted in an increased B-cell death and reduced IgG levels when cells were sorted from HIV-infected individuals (76). Impaired Tfh cell function in HIV-1 patients depends on the binding of PD-1 from Tfh cells to PD-L1, which is overexpressed on GC B cells. This interaction results in a decreased ICOS, IL-21, IL-4, and IL-10 expression by Tfh cells.

Noteworthy, in these studies, Tfr cells were not distinguished from Tfh cells and may, therefore, participate to the disturbed Tfh cell function. This question was approached in recent works studying more specifically Tfr cells that have been demonstrated to be increased in chronic HIV infection (77, 78). This expansion was related to an increase of immature DC proportion and IDO secretion favoring Tfr cell differentiation. Tfr cells from HIV-1 patients were shown to express more regulatory molecules, such as CTLA-4, LAG-3, GITR, IL-10, and TGF-β. Moreover, Tfr cell removal from follicular T-cell culture partially restored ICOS expression, IL-4, and IL-21 production, and cell proliferation of Tfh cells. Thus, Tfr cells are another contributor of inefficient GC response in HIV individuals (77).

Hypergammaglobulinemia is classically found in chronic HIV infection. It has been associated with Tfh cell amplification, as a correlation between BCL-6 in Tfh cells and total IgG and IgG1 antibody serum levels have been highlighted (70). Nevertheless, highly potent broadly neutralizing antibodies able to invalidate the majority of globally circulating HIV strains develop only in around 20% of HIV-1 infected patients (79). Broadly neutralizing antibodies against HIV display extensive somatic hypermutations (80), hypothesizing that adequate GC response does not occur in the majority of HIV-infected patients. Generation of such antibodies is one goal for HIV vaccine research. Impairment of antibody production is not restricted to anti-HIV antibodies but also concerns other T-cell-mediated antibody responses, as those elicited by exposure to neo-antigen or recall vaccination (81–83). Interestingly, highly active antiretroviral therapy is able to somehow restore adaptive immune response related to vaccination (84).

Concerning circulating Tfh cells, blood memory PD-1^+^ CXCR5^+^ CD4^+^ cell frequency is correlated with the development of broadly neutralizing antibodies against HIV (85). In addition, their absolute number is increased in patients with lower viral load and spontaneously controlling disease progression, compared to patients with high uncontrolled viremia (86). Furthermore, higher frequency of circulating Tfh cells has been associated with protective antibody responses in vaccine trials (87). Collectively, these reports hypothesize that circulating Tfh cell frequency might be used as a blood biomarker of good prognosis in HIV-infected patients. Nevertheless, additional studies using a common circulating Tfh phenotype and including a high number of patients should now be conducted.

Altogether, these studies demonstrate an impairment of the adaptive immune response in HIV seropositive patients impacting individual immune protection.

### SLO Stromal Cells

Lymphoid stromal cells are strongly affected by viral infections and contribute both to the initiation and amplification of anti-viral immune response within GCs and to virus-related immunosuppression. FDCs, the lymphoid stromal cell subset involved in the recruitment, selection, and survival of GC B cells, are now considered as a reservoir for HIV. Complement-opsonized HIV is internalized by human FDCs and retained for extensive periods in a non-degradative cycling compartment. In particular, HIV^+^ FDCs could be detected in patients under antiretroviral therapy (ART). Infectious virus could then be transmitted to uninfected CD4^+^ T cells, in particular Tfh cells, a process that could be involved in ART escape (88). Importantly, HIV pro-viral DNA is never found within FDC genome indicating that FDCs are a source of infection for T cells but are not infected by HIV. Conversely, FDCs could be infected and killed by arboviruses; thus, hampering the capacity of GCs to produce protective antibodies and inducing a transient generalized immunosuppression (89). How this mechanism, recently demonstrated for bluetongue virus in sheep, could be involved in the pathogenesis of hemorrhagic fevers caused by arboviruses in human remains to be elucidated.

Fibroblastic reticular cells are the lymphoid stromal cell subset involved in immune cell recruitment, motility, interaction, and homeostasis within lymph nodes of which they regulate the size and microenvironmental structure (13). Different viral pathogens induce distinct patterns of FRC expansion and activation, yet all induce sustained remodeling that alters responses induced by a subsequent infection (90). Importantly, FRCs are a direct target of several viruses. Ebola, Lassa, and Marburg viruses (91, 92), but also lymphocytic choriomeningitis virus (93) infect and destroy FRC network leading to lymph node disorganization and crippling the immune response to new antigens. Moreover, in chronic infection, in particular HIV, stimulation of the TGF-β receptor on FRCs by TGF-β produced by expanded Tregs leads to overproduction and deposition of collagen, causing lymph node fibrosis and restricting access of naive lymphocytes to FRC-dependent survival signals. FRC dysfunction is now considered as a major cause of HIV-related CD4^+^ T-cell depletion that in turn deprives FRCs of the lymphotoxin-β receptor signals required for their maintenance and leads to a broad immunosuppression (94). Interestingly, restoration of the FRC network and reconstitution of naive T-cell populations are only optimal when therapy is initiated in the early/acute stage of infection.

To date, very few reports decipher direct and indirect impact of viruses on FRC and FDC biology. Additional studies focusing on stromal cell and virus interactions might improve our knowledge on viral pathogenesis.

## Virus Involvement in Development and Maintenance of GC-Derived Lymphomas

Lymphomas constitute a large group of cancer arising from lymphoid or extra-nodal tissues. The nomenclature of these neoplasms regularly evolves, and currently comprises more than 50 distinct clinical, pathological, genetic, and molecular entities (95). For a subgroup of B-cell lymphomas observed in immune-competent patients and including diffuse large B-cell lymphoma (DLBCL), follicular lymphoma, classical Hodgkin lymphoma (HL), and BL, the cell of origin is located in the GC, where B cells can be infected by EBV. Virus-associated lymphomas also occur in immune-deficient patients. In this case, additionally to the previously cited entities, other lymphomas arising from GC B cells are observed, including the post-transplantation lymphoproliferative disease (PTLD) and the primary central nervous system lymphoma (PCNSL), frequently associated with HIV infection (96). These virus-associated lymphomas were recently reviewed (97, 98). Additional virus-associated lymphomas with a cell of origin outside the GC (e.g., primary effusion lymphoma) are described. The three types of EBV latency previously described are associated with various B-cell lymphomas. Type I latency is associated with BL, whereas type II is found in HL, and finally type III is found in PTLD (99).

Burkitt’s lymphoma is an aggressive B-cell lymphoma categorized in endemic, sporadic, and immunodeficiency variants. Translocation of the proto-oncogene *c-MYC* to the Ig heavy or light chain region is the hallmark of this lymphoma. The endemic variant is observed in equatorial Africa where it represents the most common malignancy of childhood, whereas the sporadic variant is seen worldwide with a median age of 30 years. Sporadic BL cases account for 1–2% of all lymphomas in Western Europe and USA. EBV is detected in almost all cases of endemic variant contrasting with the 30% of incidence in the sporadic cases and 25% in immune-deficient patients. EBV is not associated with all BL cases, suggesting that the virus is not directly involved in the pathogenesis but rather acts as a cofactor. However, the role of EBV is not clearly understood. In endemic and immunodeficiency BL, a long-term antigenic stimulation by bacteria, virus, or parasite (in particular malaria) precedes the lymphoma and may induce an exhaustion of T cells and, thus, a defect in response to infected B cells (100, 101). In addition, chronic antigen stimulation in the GC might enhance the likelihood of c-MYC Ig rearrangement (98).

Hodgkin lymphoma is defined by an expansion of Reed–Sternberg cells within a reactive microenvironment. There are classified as nodular lymphocyte predominant HL (NLPHD) and classical HL (cHL). Within cHL, four subtypes are recognized. These subgroups differ clinically and also in frequency of EBV infection ranging from 10 to 40%. Noteworthy, EBV is rarely associated with NLPHD. LMP2 is involved in rescuing Reed–Sternberg cells, which commonly present an aberrant BCR, from apoptosis. LMP1 also participates in the constitutive activation of signaling pathways (e.g., NF-kB, JAK/STAT) in Reed–Sternberg cells (101, 102).

Diffuse large B-cell lymphoma is the most common non-HL, accounting for around 40% of new cases. Although the World Health Organization recognizes DLBCL as a single entity, several subgroups with different outcomes have been described (96). All subtypes are associated with various frequencies of EBV although high rates are associated with immune deficiency lymphomas. Recently, a subtype called “EBV^+^ DLBCL not otherwise specified (NOS)” has been recognized in immune-competent patients (95). They have a worse prognosis than their EBV^-^ counterparts. The role of EBV is not clearly understood in this disease where LMP-1 is detected in almost all cases, but EBNA2 only in a minority of patients (98). PCNSL is an aggressive DLBCL occurring in immune-deficient patients, mainly in HIV infection, and is associated with EBV in all cases. LMP1 and EBNA2 are expressed in tumor cells (97).

Post-transplantation lymphoproliferative disease is a heterogeneous group of lymphomas occurring after allogeneic transplantation of solid organ or hematopoietic stem cell graft. A B-cell phenotype is observed in 85% of cases. In 65% of PTLD, a reactivation of EBV occurs because anti-EBV immunity is impaired by the immunosuppressive therapy following the transplantation (103).

Epstein–Barr virus can also be associated with T-cell and NK-cell lymphomas. Among them, angioimmunoblastic T-cell lymphoma is a rare malignancy primarily involving lymph nodes, and characterized by tumor T cells expressing CXCL13, CD10, PD-1, and BCL-6, such as Tfh cells, their normal counterpart (20, 104). EBV involvement in lymphoma development and maintenance is unclear. Nevertheless, EBV is carried by lymph node B cells and in rare tumor and infiltrating non-tumor T cells (105).

Finally, viruses can also act on the tumor microenvironment (99). As reported previously, HIV infection induces an impairment of the adaptive immune response. In addition, EBV-encoded proteins have been demonstrated to increase expression and release of cytokines, such as IL-6 or IL-10, that may impact tumor B-cell growth (106).

Epstein–Barr virus proteins can also induce enhanced expression of vascular endothelial growth factor, IL-8, or hypoxia-inducible factor-1α, all contributing to angiogenesis, which is an important mechanism of tumor growth (107, 108). Moreover, exosomes derived from EBV-infected cells may also contribute to tumor growth by apoptosis induction of CD4^+^ EBV-specific T cells, Th1, Th17, and T CD8^+^ cells, as well as expansion of Treg cells (99, 109). Interestingly, EBV triggers high PD-L1/CD274 expression on malignant cells in both DLBCL, cHL without amplification of the chromosomal region 9p24.1 that contains the genes encoding both PD-L1 and PD-L2, and PTLD (110). PD-L1 upregulation has been associated with exhaustion of tumor infiltrating T cells and tumor escape suggesting that EBV infection induces PD-L1 expression on lymphocytes in order to promote a tolerogenic immune state. In conclusion, EBV is associated with various B-cell and T-cell lymphomas arising from GC in immune-competent or immune-deficient hosts. So far the pathogenesis is not clearly understood and several mechanisms may be involved. EBV can trigger the tumor cell directly such as interfering with signaling pathways or promoting amplification of oncogenes, or acts indirectly by impairing antitumor response (summarized in Table 1).

**Table 1:** Virus-associated germinal center (GC)-derived lymphomas.

Disease	Virus	% of involvement	Mechanism
Burkitt’s lymphoma (BL)	Epstein–Barr virus (EBV) (type I latency)	30% for sporadic BL; 100% for endemic BL; 25% in immune-deficient patients	Cofactor? Defect in response to infected B cells; Chronic GC stimulation enhance c-MYC rearrangement

Hodgkin lymphoma (HL)	EBV(type II latency)	10–40% for classical HL (cHL) subgroups; Rarely associated with nodular lymphocyte predominant HL	Rescuing Reed–Sternberg cells from apoptosis (LMP2) Constitutive activation of signaling pathways (LMP1)

Diffuse large B-cell lymphoma (DLBCL)	EBV (type II or III latency)	100% in EBV + DLBCL not otherwise specified	LMP1 + EBNA2−
	EBV and HIV	Immune-deficient patients: primary central nervous system lymphoma	LMP1 + EBNA2+

Post-transplantation lymphoproliferative disease	EBV (type III latency)	>90%	In 65% of cases, reactivation of EBV after immune suppressive treatment

## Conclusion

Like during other maturation stages, B and T cells in GCs can be targeted by virus infection. Accumulating reports demonstrate that GC B-cell and T-cell infections disturb specific adaptive immune responses and vaccination efficiency, and can worsen a pre-established autoimmune disease, or participate to cancer development and maintenance. In addition, recent pieces of evidence elucidate that viruses can also infect GC stromal cells, causing a stroma network disorganization leading to disturbed humoral immunity. Altogether, this underlines that virus infection of all GC cell-components must be take into account to understand impact of viruses on the T-cell dependent humoral immune response. Moreover, a better characterization of the functional consequences of chronic viral infection on various GC cell subsets paves the way for the design of new efficient therapeutic strategies in different pathological context. In particular, PD-L1^hi^ EBV^+^ HL and DLBCL tumor cells may be suitable therapeutic targets for anti-PD-1/PD-L1 immunotherapy with the aim to unleash host antitumor immune responses. Moreover, the recently identified roles of Tfh cells, FDCs, and FRCs as HIV-1 reservoirs or targets, potentially involved in early and late relapse to ART, could provide new prognosis biomarkers and therapeutic targets, including the use of antifibrotic molecules to revert HIV-1-induced damage to lymphoid stromal cell niches, the purging of the FDC reservoir, or the targeting of infected Tfh cells (111).

## Author Contributions

SR, MR, KT, and PA are substantial contributors to the conception of this work.

## Conflict of Interest Statement

The authors declare that the work was conducted in the absence of any commercial or financial relationships that could be construed as a potential conflict of interest.
